# An Estimate of Avian Mortality at Communication Towers in the United States and Canada

**DOI:** 10.1371/journal.pone.0034025

**Published:** 2012-04-25

**Authors:** Travis Longcore, Catherine Rich, Pierre Mineau, Beau MacDonald, Daniel G. Bert, Lauren M. Sullivan, Erin Mutrie, Sidney A. Gauthreaux, Michael L. Avery, Robert L. Crawford, Albert M. Manville, Emilie R. Travis, David Drake

**Affiliations:** 1 The Urban Wildlands Group, Los Angeles, California, United States of America; 2 Spatial Sciences Institute, University of Southern California, Los Angeles, California, United States of America; 3 National Wildlife Research Centre, Science and Technology Branch, Environment Canada, Ottawa, Canada; 4 Department of Geography, University of California Los Angeles, Los Angeles, California, United States of America; 5 Department of Biological Sciences, Clemson University, Clemson, South Carolina, United States of America; 6 National Wildlife Research Center, Florida Field Station, United States Department of Agriculture/Wildlife Services, Gainesville, Florida, United States of America; 7 Thomasville, Georgia, United States of America; 8 Division of Migratory Bird Management, United States Fish and Wildlife Service, Arlington, Virginia, United States of America; 9 Department of Forest and Wildlife Ecology, University of Wisconsin, Madison, Wisconsin, United States of America; University of Otago, New Zealand

## Abstract

Avian mortality at communication towers in the continental United States and Canada is an issue of pressing conservation concern. Previous estimates of this mortality have been based on limited data and have not included Canada. We compiled a database of communication towers in the continental United States and Canada and estimated avian mortality by tower with a regression relating avian mortality to tower height. This equation was derived from 38 tower studies for which mortality data were available and corrected for sampling effort, search efficiency, and scavenging where appropriate. Although most studies document mortality at guyed towers with steady-burning lights, we accounted for lower mortality at towers without guy wires or steady-burning lights by adjusting estimates based on published studies. The resulting estimate of mortality at towers is 6.8 million birds per year in the United States and Canada. Bootstrapped subsampling indicated that the regression was robust to the choice of studies included and a comparison of multiple regression models showed that incorporating sampling, scavenging, and search efficiency adjustments improved model fit. Estimating total avian mortality is only a first step in developing an assessment of the biological significance of mortality at communication towers for individual species or groups of species. Nevertheless, our estimate can be used to evaluate this source of mortality, develop subsequent per-species mortality estimates, and motivate policy action.

## Introduction

On the morning of September 11, 1948, “a good number of dead, dying, and exhausted birds” were found at the base of the WBAL radio tower in Baltimore, Maryland [1]. Reports of such avian mortality at communication towers in North America became common in the 1950s [2]–[7]. These observations were consistent with the long documented mortality of birds at lights, including lighthouses [8], light towers [9], buildings [1], [10], and ceilometers [1], [11]. Although initially dismissed as being of minor consequence [12], the ongoing and chronic mortality of nocturnally migrating birds at lighted structures has become a recognized conservation issue [7], [13]–[15]. Bats are also killed in collisions with tall towers in unknown numbers [16]–[18]. An estimate of the total number of birds killed at communication towers in the United States and Canada is particularly relevant because the current transition from analog to exclusively digital broadcasting in the United States is expected to lead to the construction of more tall towers and a similar trend will likely follow in Canada.

In 1979, Banks [13] developed a widely circulated estimate of avian mortality at television towers, which revised upward a previous estimate by Mayfield [12]. In Banks’s assessment of various sources of human-caused avian mortality, he extrapolated the results of three studies at tall towers – two in Florida [19], [20] and one in North Dakota (for which he did not provide a citation but which was almost certainly [21]) – to all television towers. He calculated the average mortality at these three sites to be roughly 2,500 birds per year, and multiplied it by the number of television towers (1,010 in 1979). He then assumed that half of all television towers would cause a hazard to migrating birds. The resulting estimate of annual mortality was 1,250,000 [13]. Also in 1979, Avery [22] applied bird mortality results from seven towers that had been monitored for at least 10 years and derived an overall mortality estimate of 940,000/year for the United States. More recent estimates of total avian mortality at towers in the United States by the U.S. Fish and Wildlife Service (USFWS) in 2001 [14], [23] adjusted the Banks estimate by accounting for the increased number of towers since 1979. Application of Banks’s method today results in an estimate of 4–5 million birds killed annually by tall towers, with Manville [15], [24] indicating a possibility of mortality an order of magnitude higher.

No estimate of avian mortality at communication towers has been made for the United States and Canada as a whole, and the only estimate for Canada was presented in a preliminary unpublished report preceding this paper. The bulk of species killed at towers in the United States and Canada are Neotropical migrants, i.e., birds that breed in Canada and the United States and spend the non-breeding period south of the U.S. border [13], [25]. Because the ranges of these species extend into Canada, mortality in both the United States and Canada contribute to their population dynamics.

In this paper we develop a new estimate of avian mortality at communication towers in the United States and Canada. This estimate derives from a review and re-analysis of tower mortality studies (following [26]). We improve on Longcore et al. [26] by adjusting mortality records at towers for sampling effort, search efficiency, and scavenging, and by incorporating additional studies. We produced a regression for avian mortality by tower height and then applied this regression to a geographic database of communication towers for the United States and Canada. This approach recognizes that taller towers kill more birds on average than do shorter towers [26]–[28], but also incorporates mortality estimates for lighted towers that are less than 600 ft (∼180 m) above ground level (AGL), which have previously been left out of estimates of total avian mortality. These “shorter” (60–180 m) lighted towers, which constitute >95% of lighted towers, do regularly kill birds [28]–[30] and their sheer number argues against ignoring them. We do not, however, estimate mortality from collisions with other lighted structures. Attraction to light at night leads to avian mortality at buildings, monuments, cooling towers, bridges, offshore platforms, ships, lighthouses, and wind turbines [24], [31], [32], and the same group of species (Neotropical migrants) are especially susceptible.

Our goal is to improve upon past estimates, which relied on a very limited set of data and did not reflect current understanding of the tower height–mortality relationship. Because of the nature of the existing data on avian mortality at towers and the lack of a systematic continent-wide survey effort, additional field studies will be required to refine further our approach. Our results do, however, increase both the transparency and accuracy associated with the estimate of this source of avian mortality.

## Methods

We assigned average mortality values to tower height classes (every 30 m) using a regression of tower height by annual mortality (following [26]). Longcore et al. [26] identified reports of birds killed at 26 communication towers over at least two migratory seasons (e.g., spring and fall, two falls), consisting of a minimum of 10 total carcass-searching visits per site. We added figures from additional studies [33], [34], tested the sensitivity of the regression to inclusion of studies, and developed adjustments for sampling effort, search efficiency, and scavenging to produce estimates of mortality.

### Sensitivity of Tower Height–mortality Regression

We collected as many studies of bird mortality at communication towers as possible from the literature and, when necessary, obtained raw data from study authors. Some studies had to be dropped from our analysis (e.g., [28]) because we were unable to obtain data from study authors and published reports did not allow us to assign mortality to specific towers. Because the number of tower studies available to us was finite, and because the choice of studies may have influenced our results, we tested the extent to which the regression was robust to sampling variation among the towers available for analysis. We used a randomization and resampling procedure to select random subsets of the 38 towers included in the analysis. To explore a range of plausible tower subsets that could produce a regression, we resampled subsets that included just under half of the available towers (18) up to those with one fewer than the complete dataset (37 towers) and re-iterated the sampling procedure 5,000 times. We used the natural logarithm of both the dependent and independent variables to normalize their distributions.

### Adjustment for Scavenging and Search Efficiency

Loss of birds to scavengers and failure to detect all dead birds (search efficiency) are sources of error and variation in tower studies. Some authors have opted to apply searching and scavenging factors to final kill estimates (e.g., [28], [35]). Recognizing that search efficiency and scavenging losses are likely tower-specific, we opted to correct the number of kills at each tower before regressing these estimated losses against tower height.

We assumed that scavenging would be lower at a small tower that sporadically generates only a few mortalities compared with a well-established tall tower that kills birds reliably and therefore maintains scavenger interest [36]–[39]. This assumption is supported by high scavenging rates documented at tall towers such as WCTV in Florida [20], [36], [38] and rapid increases in scavenging when researchers provide carcasses [33]. Even with extensive scavenger control efforts, Stoddard estimated that he was losing at least 10% of bird carcasses to scavengers daily [40]. Therefore, we adjusted our scavenging rate by tower height.

We assumed that it is easier to find carcasses under a short tower because carcasses are likely to be less dispersed under shorter guy wires or in the absence of guy wires. Whether the area around the tower is bare or heavily vegetated will affect both scavenging and search rates [41]. Open habitats with little concealing vegetation are, predictably, more conducive to efficient searching for carcasses [41]. Scavengers detecting prey by sight can find the carcasses more easily as well. Notwithstanding the use of smell by some carnivores to find prey, dense cover makes it more difficult in general for both scavengers and searchers to find carcasses [42]. Support for our assumptions about the effect of cover on these rates is found in research on avian mortality caused by pesticides, power lines, and wind turbines [41]–[45]. We avoided attempts to calculate probability of detection by searchers that involved the “life expectancy” of carcasses because these methods are biased [46]. If a carcass was not found on the first search day, the probability that it will be found on subsequent days is considerably less than the average search rate would suggest. Therefore, for the purpose of this analysis, the likelihood that a carcass was found more than one day after it was generated is considered negligible. Removal of dead birds by scavengers at sites with regular mortality also follows an exponential decay model such that the probability of small dead birds remaining to be found falls quickly following the mortality event [45], [47].

We divided towers into height classes to which we could assign differential search and scavenging rates. Based on breaks in the raw tower mortality data, we chose to divide the towers into three height classes: 0–200 m, 201–400 m, and ≥401 m. To assign search and scavenging rates we relied on our published summaries of available rates from a range of carcass searching contexts (Table 1) [41], [42], other existing studies and reviews [37], [43], [44], [46], [48], and values reported at the towers in our dataset where these rates were measured [28], [33], [34], [49]. Taking into account patterns from these data, we used tower height as well as any information about cover as a way to assign search and scavenging corrections by height and cover class to the towers for which these rates had not been measured and reported by the authors (Table 2). All search and scavenging rates, both measured and assigned, are reported in Table 3.

**Table 1:** Average search and scavenging rates taken from pesticide impact studies [42].

Habitat	Body size	Search rate (# study plots)	Percentage lost to scavenging	Detection rates (studies combining search and scavenging rates)
Shrub/wood edge	Small-medium	41.0% (301)	20.9%	22.8% (94)
Shrub/wood edge	Large	67.6% (29)	-	-
Bare/open	Small-medium	64.6% (359)	28.4%	18.6% (56)
Bare/open	Large	88.1% (17)	-	-

Search and detection rates are based on daily averages weighted by the number of study plots. Search rates represent the proportion of carcasses found over the total number still present at the time of search. Scavenging rates represent daily measurements averaged over all plots without regard for the number of placed carcasses. Search rates are undoubtedly at the high end of that which is possible because the search procedures were optimized, always including trained lines of searchers spaced optimally for the habitat as well as the use of search dogs in some studies.

**Table 2:** Assumed rates for search efficiency and scavenger removal by tower height and habitat type when not provided by investigator.

Tower type and mortalityprofile	Habitat	Assumed proportion of small birds located by searcher	Assumed proportion ofsmall birds remainingafter scavenging	Combined rate of detection
Height class 1 (0–200 m), sporadic mortality, more localized	Open habitat	75%	80%	60%
	Brush and other visual obstructions	50%	85%	42%
Height class 2 (201–400 m), regular mortality, more dispersed	Open habitat	65%	55%	36%
	Brush and other visual obstructions	40%	70%	28%
Height class 3 (≥401 m),dependable mortality, carcasseswidely dispersed	Open habitat	55%	30%	16%
	Brush and other visual obstructions	30%	55%	16%

**Table 3:** Summary of factors used to develop the search and scavenging correction for bird mortality at communication towers.

Reference	Cover	Daily	Tower height (m)	Scavenger control	Scavenging measured	Search efficiency measured	Measured or assumed search rate	Measured or assumed scavenging rate	Overall detection rate
[69]	burned spring, hayed fall	No	30.5	no	no	no	0.750	0.200	0.600
[49]	cleared periodically	No	60	yes	yes	yes	0.406	0.392	0.247
[34]	mowed at least once per season	Yes	60	no	yes	yes	0.294	0.076	0.271
[34]	mowed at least once per season	Yes	60	no	yes	yes	0.294	0.076	0.271
[34]	mowed regularly	Yes	79	no	yes	yes	0.294	0.076	0.271
[40]	Mowed	Yes	90	yes	no	no	0.750	0.100	0.675
[34]	mowed at least once per season	Yes	97.5	no	yes	yes	0.290	0.113	0.257
[34]	mowed regularly	Yes	108.5	no	yes	yes	0.290	0.113	0.257
[34]	mowed regularly	Yes	110.3	no	yes	yes	0.290	0.113	0.257
[70]	bare ground and pavement under tower, weeds/grasses elsewhere	No	133	no	no	no	0.750	0.200	0.600
[34]	mowed regularly	Yes	141.7	no	yes	yes	0.380	0.213	0.299
[34]	alfalfa field, mowed infrequently	Yes	142	no	yes	yes	0.380	0.213	0.299
[33]	rocky, some shrub	No	152	no	yes	yes	0.850	0.030	0.825
[71]	most birds measured fell on roof of building	No	161	*yes (roof)	no	no	0.750	0.200	0.600
[34]	mowed regularly	Yes	163	no	yes	yes	0.380	0.213	0.299
[3], [72]–[88]	wooded/rocky and roof of building	No	287	*yes (roof)	no	no	0.650	0.450	0.358
[89]	cut grass (cut to different lengths)/paved	yes but only in the first year 1971	293	no	no	no	0.650	0.450	0.358
[5]	corn/soybean field	No	299	no	no	no	0.400	0.300	0.280
[90]	unknown (probably open or mowed)	No	300	no	no	no	0.650	0.450	0.358
[91]	Open	Yes	305	no	no	no	0.650	0.450	0.358
[40]	Mowed	Yes	308	yes	no	no	0.650	0.100	0.585
[89]	cut grass (cut to different lengths)/paved	yes but only in the first year 1971	323	no	no	no	0.650	0.450	0.358
[89]	cut grass (cut to different lengths)/paved	yes but only in the first year 1971	328	no	no	no	0.650	0.450	0.358
[89]	cut grass (cut to different lengths)/paved	yes but only in the first year 1971	330	no	no	no	0.650	0.450	0.358
[90]	unknown (probably open or mowed)	No	342	no	no	no	0.650	0.450	0.358
[92]	dirt, weedy sand, grass/low weed under guy wires, dense vegetation everywhere else	No	362	no	no	no	0.400	0.300	0.280
[21], [50], [51]	Dense	Yes	366	no	yes	no	0.400	0.100	0.360
[93]	Unknown	No	366	no	no	no	0.650	0.450	0.358
[90]	unknown (probably open or mowed)	No	390	no	no	no	0.650	0.450	0.358
[34]	mowed regularly	Yes	395.5	no	yes	yes	0.294	0.332	0.197
[94]	mostly pasture but also pavement and bare ground directly under tower	No	400	no	no	no	0.650	0.450	0.358
[95], [96]	mowed at least once	No	411	no	no	no	0.550	0.700	0.165
[73], [74], [76]–[88], [97]–[100]	unknown but open	yes fall only	417	no	no	no	0.550	0.700	0.165
[34]	higher grasses, mowed infrequently	Yes	433.7	no	yes	yes	0.294	0.332	0.197
[101]	burned spring, hayed fall	No	439	no	no	no	0.550	0.700	0.165
[19], [102]	water and unvegetated ground/dirt	No	452	no	no	no	0.550	0.700	0.165
[92]	dirt, weedy sand, grass/low weed under guy wires, dense vegetation everywhere else	No	608	no	no	no	0.300	0.450	0.165
[103]	“heavy” ground cover	No	610	no	no	no	0.300	0.450	0.165

We investigated the sensitivity of our final results to these assumptions about search efficiency and scavenging by recalculating our total mortality estimates while assigning the average search efficiency and scavenging rates reported from those studies that did estimate these rates. This approach tested the alternative assumption that studies from all towers where search efficiency or scavenging were not measured had the same search efficiency, scavenging rate, or both, as did studies at the towers where they were measured, regardless of the physical conditions at the tower or the height of the tower.

### Adjustment for Sampling Effort and Design

**Figure 1:**
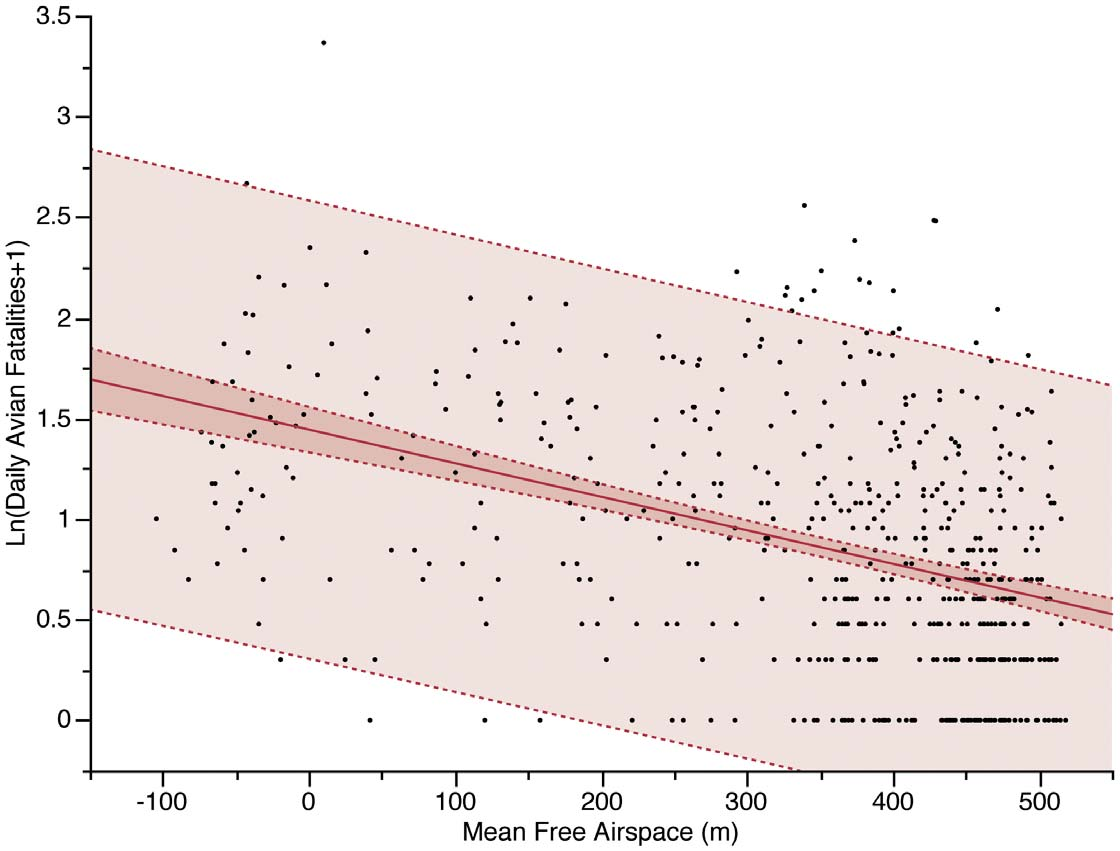
Relationship of bird fatalities to free airspace at WCTV Tower, 1956–1967. Raw data from Crawford and Engstrom (2001) were used to plot daily bird fatalities against the mean free airspace between the top of the tower and the cloud ceiling each day. Days with maximum ceiling were excluded. Daily avian mortality increases significantly as free airspace decreases (Ln(Bird Fatalities +1) = 1.443928 – 0.0016667 · Mean Free Airspace (m), *R* ^2^ = 0.17, p<0.001).

Studies included in the tower height–mortality regression varied in sampling design and duration. Following Longcore et al. [26], we required a minimum of 10 searches for a study to be included. Authors of most of the studies used in the regression assumed that most birds would be found by sampling during peak migration, on bad weather days preceding or following the passing of a cold front (e.g., J. Herron, pers. comm.), or both (Table 4). The logic behind this approach is that many high avian mortality days are correlated with these factors [31]. Nevertheless, “trickle kills” on fair weather days even outside the typical migration period can contribute substantially to overall mortality [40]. Substantial mortality during clear and calm weather during the migration season has also been documented [30], [50] (Figure 1). For these reasons we used raw data from two studies that carried out daily carcass searches – WCTV Florida tower data from 1956–1967 initiated by Herbert L. Stoddard and Tall Timbers Research Station [40] and North Dakota “Omega” tower [21], [51], [52] – as a baseline to develop estimates of the effectiveness of the various sampling designs for the 38 tower studies included in our dataset. The Florida estimates were averaged over the 10 years of sampling during which height of tower and predator control were the same; the North Dakota estimates are for two years of sampling. When the estimate was (partially) based on sampling outside the migratory period (as defined), we used the Florida dataset, which had continuous, year-round sampling. We did not, however, correct upward all kill estimates to account for the trickle of kills recorded in the non-migratory seasons. We believe, therefore, that our estimates are conservative. To control for differences between spring and fall migration we developed estimates for both spring and fall separately.

**Table 4:** Summary data with sampling efficiency correction for the 38 studies used to develop an estimate of bird mortality at communication towers.

Reference	Tower height (m)	Start year	End Year	Sampling days	Sampling correction	Sampling strategy	No. of years	Average correction sampling (spring)	Average correction sampling (fall)	Birds collected	Mean annual fatalities (raw)	Mean annual fatalities (corrected sampling and scavenging)
[69]	30.5	1998	1999	25/year	yes	bad weather	1	0.44	0.36	0	0.0	0.0
[49]	60	2000	2004	average >70/year	yes	bad weather	4	0.50	0.50	15	3.8	30.4
[34]	60	2007	2008	45 spring, 45 fall	no	n/a	2	1.00	1.00	3	1.5	5.5
[34]	60	2007	2008	45 spring, 45 fall	no	n/a	2	1.00	1.00	1	0.5	1.8
[34]	79	2007	2008	45 spring, 45 fall	no	n/a	2	1.00	1.00	8	4.0	14.8
[40]	90	1998.5	2000	>330/year	no	n/a	1.5	1.00	1.00	21	14.0	20.7
[34]	109	2007	2008	45 spring, 45 fall	no	n/a	2	1.00	1.00	7	3.5	13.6
[34]	110	2007	2008	45 spring, 45 fall	no	n/a	2	1.00	1.00	6	3.0	11.7
[34]	110	2007	2008	45 spring, 45 fall	no	n/a	2	1.00	1.00	3	1.5	5.8
[70]	133	1958	1960	<60/year	no	n/a	2	1.00	1.00	267	133.5	222.5
[34]	142	2007	2008	45 spring, 45 fall	no	n/a	2	1.00	1.00	14	7.0	23.4
[34]	142	2007	2008	45 spring, 45 fall	no	n/a	2	1.00	1.00	5	2.5	8.4
[33]	152	2004	2006	>52/year	yes	bad weather + weekly	2	0.90	0.58	11	5.5	10.5
[71]	161	1980	1986	15.25/year average	yes	bad weather	6	0.44	0.36	700	116.7	515.6
[34]	163	2007	2008	45 spring, 45 fall	no	n/a	2	1.00	1.00	20	10.0	33.4
[3], [72]–[88]	287	1956.5	1973	<60/year	no	n/a	18.25	1.00	1.00	6470	354.5	991.7
[89]	293	1969	1999	unknown	yes	bad weather	30	0.44	0.36	8011	267.0	1998.4
[5]	299	1955	1957	7 confirmed	yes	big kills	2	0.66	0.79	486	243.0	1149.1
[90]	300	1959.5	1964	unknown	yes	bad weather	4.5	0.44	0.36	199	44.2	330.9
[91]	305	1957	1995	>180/year	no	n/a	38	1.00	1.00	121560	3198.9	8948.1
[40]	308	1970	1983	>330/year	no	n/a	13	1.00	1.00	8035	618.1	1056.5
[89]	323	1969	1999	unknown	yes	bad weather	30	0.44	0.36	1043	34.8	260.2
[89]	328	1969	1999	unknown	yes	bad weather	30	0.44	0.36	11092	369.7	2766.9
[89]	330	1973	1992	unknown	yes	bad weather	19	0.44	0.36	4310	226.8	1697.6
[90]	342	1958.8	1964	unknown	yes	big kills	5.25	0.66	0.79	1740	331.4	1227.5
[92]	362	1970	1972	unknown	yes	bad weather	2	0.44	0.36	995	497.5	4753.6
[21]	366	1972	1974	>180/year	no	n/a	2	1.00	1.00	785	392.5	1090.3
[93]	366	1962.5	1964	12 spring, 12 fall	yes	bad weather	1.5	0.44	0.36	125	83.3	623.6
[90]	390	1958.8	1964	unknown	yes	overcast	5.25	0.44	0.36	3972	756.6	5661.8
[34]	396	2007	2008	45 spring, 45 fall	no	n/a	2	1.00	1.00	760	380.0	1931.7
[94]	400	1969	1974	<10/year	yes	big kills	5	0.66	0.79	3507	701.4	2597.8
[95], [96]	411	1969.5	1971	unknown	yes	bad weather	1.5	0.66	0.79	508	338.7	2717.7
[73], [74], [76]–[88], [97]–[100]	417	1960.5	1997	<60 year	no	n/a	27.5	1.00	1.00	20192	734.3	4450.0
[34]	434	2007	2008	45 spring, 45 fall	no	n/a	2	1.00	1.00	237	118.5	602.4
[101]	439	1999	2000	18 spring, 32 fall	yes	bad weather	2	0.44	0.36	946	473.0	7669.4
[19], [102]	452	1969	1972	>5/year	yes	big kills	3	0.66	0.79	9130	3043.0	24421.8
[92]	608	1970	1972	unknown	yes	overcast	2	0.44	0.36	2223	1111.5	18022.3
[103]	610	1973.3	1975	unknown	yes	overcast day pairs	1.75	0.44	0.36	3521	2012.0	32623.3

Number of years in each study may differ from the calendar years encompassed by the study because of the assumption that each fall constitutes 0.75 years of surveying and each spring constitutes 0.25 years of surveying. Studies in which surveys were conducted only during the fall or only sporadically during the spring will appear to be shorter than their calendar duration.

To adjust for the kills between sampling days during the migratory seasons we resampled (with replacement) daily mortality data from the Florida and North Dakota datasets within each of the spring and fall migration periods by randomly selecting a subset of days and summing avian mortality for the selected days. We calculated average bird mortality for 5,000 iterations and then used the ratio of the average bird mortality from the 5,000 iterations to the total number of birds killed during either spring or fall migration or outside of the migration period to adjust mortality estimates for studies without daily sampling. We averaged estimates between the Florida and North Dakota datasets. This adjustment was applied to studies where researchers sampled on bad weather days (see below) and to those with weekly sampling outside the migration period.

For studies that did not provide complete details on their sampling design, we made simplifying assumptions (see below). If more than one sampling strategy was used, we developed estimates for each and used the sum as our overall estimate. For example, sampling may have been done weekly (regular sampling) outside of the migration period and also on “bad weather days” during the migration period.

We defined the spring and fall migration periods as a 60-day window before and after the migration peak for both spring and fall for each dataset, recognizing that for some recent studies (e.g., [28]) monitoring only occurred during the three-week peak of migration. We determined the peak for the Florida and North Dakota datasets by plotting the number of birds killed (from the raw data) against Julian date for all years of data combined and using negative exponential smoothing.

Some investigators reported the total number of days sampled during one or both migration periods and sometimes outside the migration periods. When the sampling interval (e.g., weekly) was identified in the study design, we constrained the resampling procedure to randomly select a day within that sampling interval. If no sampling interval was defined, selection was random.

Some investigators sampled on so-called “bad weather days” or following bad weather nights, i.e., overcast, often associated with advancing cold fronts and potentially including precipitation. Usually no other information was provided to define bad weather or the number of days when bad weather occurred. High bird mortality at communication towers is correlated with bad weather days [40], [50], [53]. This is shown by plotting ln(n+1)-transformed daily mortality data from the Florida tower dataset for the 1956–1967 fall migrations against the mean free airspace (distance between the top of the tower and the bottom of the cloud cover). Days where maximum free airspace was recorded were excluded from analysis because measurements did not vary for total ceiling greater than 610 m (2,000 ft). Mortality for days with mean ceiling at the maximum was 4.0–8.0 birds per day (95% C.I., n = 871), while mortality for all days with less than the maximum ceiling was 16.0–33.5 birds per day (95% C.I., n = 569). Considering these remaining points, a linear regression reveals a highly significant effect of mean free airspace, but also low explanatory power (Figure 1). Based on these data, we used days with mean free airspace equal to or below 335 m (1,100 ft) as an index of bad weather days because mortality was significantly lower on days with airspace greater than 335 m (10.3–17.8 birds per day, 95% C.I., n = 387) compared with days with airspace below this threshold (21.5–73.3 birds per day, 95% C.I., n = 182).

For some studies, the only information provided was the number of days sampled and the timing of sampling (during migration or all year). For these studies we assumed that researchers sampled on bad weather days during migration when large bird kills at communication towers were expected, given that this was the response obtained when we were able to contact researchers to ask about papers where this detail was not provided (e.g., J. Herron, pers. comm.).

Several researchers sampled only on days when so called “big kills” were reported. The definitions of “big kill” were not included. The typical daily trickle of dead birds for the Florida dataset over the 1956–1967 period was five. We therefore defined big kills as six or more birds located after any given night.

We investigated the sensitivity of our results to our assumptions about sampling effort by varying these assumptions for the 13 studies in our dataset that either did not indicate the number of days sampled or did not provide a definition of sampling design, or did neither. Some researchers had indicated that they had sampled on overcast or bad weather days or following bad weather days. For all of these studies and for those that did not mention anything specific, we made the conservative assumption that towers were sampled on bad weather days. We then recalculated the sampling adjustment and total mortality using three different scenarios: 1) researchers sampled on bad weather days and weekly during migration (e.g., [49]); 2) researchers sampled on bad weather days and weekly all year (e.g., [33]; excludes 5 of the 13 studies that clearly indicated they only sampled during migration); and 3) researchers sampled only following big kill days, about which they were notified by personnel at the tower (e.g., [5]).

### Evaluation of Model Correction Factors

We plotted either raw carcass counts or mortality estimates corrected for either sampling effort or search efficiency and scavenging, or both, against tower height and looked for improvements in the regression coefficient as an indication that the corrections improved the model.

### Description of Communication Towers and their Characteristics

We used a Geographic Information System (GIS) to extract the locations and characteristics of towers in the Antenna Structure Registration (ASR) database maintained by the U.S. Federal Communications Commission (FCC) and the NAV CANADA obstruction database. The FCC data are freely available and we purchased a license for the Canadian obstruction data for the limited purpose of this study. We compared and crosschecked these with the FCC’s microwave tower database and the commercial TowerMaps database (also purchased, see http://www.towermaps.com/), which provides locations of cellular towers to potential lessees and incorporates both data for shorter towers and information that was not included in the FCC databases. We did considerable quality control on the tower data, confirming from independent sources that all towers greater than 300 m existed. This was necessary because the data were prone to multiple types of errors; for example, the FCC database included a record claiming to be located in the “Land of Oz” in Kansas, associated with geographic coordinates in Minnesota. Full details of the quality assurance are available from the authors.

The NAV CANADA database did not contain comprehensive information about either the presence of guy wires or the presence and type of lighting. We therefore relied on data from the FCC and TowerMaps datasets and assumed that lighting and guy wire use was similar in both countries for towers of the same height class, an assumption supported by the similarity in marking and lighting standards between the two countries. The U.S. Federal Aviation Administration requirements are found in the advisory circular AC 70/7460-1K. Those of Canada are found in Standard 621 of the Canadian Aviation Regulations.

### Calculation of Annual Mortality

Avian mortality was estimated with the antilogarithm of the regression of the log transformed variables, which was adjusted for transformation bias using the smearing estimator after testing to confirm homoscedasticity of variance in the regression [54], [55]. Most recorded tower kill events take place at guyed towers, and steady-burning lights increase the probability of large tower kills [26], [28]. We assumed that unguyed towers caused 85% less mortality than guyed towers (midpoint of 69–100% estimate in [56]) and that towers without steady-burning lights caused 60% less mortality than towers with such lights (midpoint of 50–71% estimate in [28]). Following Longcore et al. [26], all estimates were calculated assuming that when both seasons were not measured, 75% of annual mortality occurred during the fall and 25% during the spring [40].

We overlaid locations of towers within each Bird Conservation Region (BCR) in the study area and calculated the number of towers in each 30 m height class for all towers ≥60 m. Bird Conservation Regions are divisions defined by habitat and topography that have been delineated for the purpose of bird conservation by the North American Bird Conservation Initiative and are endorsed by a range of bird conservation organizations and government agencies. BCRs are based on the North American ecoregions developed to promote international conservation efforts [57]. For each height class within each BCR we calculated the average number of birds killed per year, using the tower height–mortality regression adjusted for sampling effort, search efficiency, and scavenging as described above. For purposes of calculating total mortality we included all towers in the continental portions of the United States and Canada. Although most literature on tower mortality in North America describes studies from east of the Rocky Mountains, we included the West as well for purposes of estimating total mortality, a decision supported by records of tower mortality in Colorado [33], New Mexico [58], and Alaska [59], in addition to documented kills at lighthouses in California and British Columbia [60], [61]. We did not attempt to assign differential mortality for so-called flyways because radar studies and other observations strongly support the existence of “broad front” migration [62], [63]. To investigate this assumption, we plotted the residuals of the tower height–mortality regression by their geographic coordinates and calculated Moran’s I as a measure of spatial autocorrelation. We acknowledge that local habitat factors may influence mortality at particular towers, but because only 18.4% of towers were originally selected for monitoring on the basis of knowledge of prior mortality (see below), it is unlikely that these variations would result in a systematic bias in the resulting mortality estimates.

To illustrate the contribution of each part of our adjustment to the final estimate of mortality, we calculated the extrapolated mortality estimates for the unadjusted mortality data, with the sampling correction only, with the search efficiency and scavenging corrections only, and corrected for all factors.

We do not report estimates of bird mortality at short (<60 m) towers in this paper because they contribute negligibly to overall annual bird mortality and are not usually illuminated unless located near an airport. We note, however, that single-night mortality events with several hundred identified dead birds at unlit <60 m towers have been reported, often related to lighting at adjacent infrastructure [30], which is consistent with reports from turbines and towers monitored at industrial wind facilities [64]. Our analysis therefore applies to towers ≥60 m.

## Results

### Tower Height–mortality Regression

**Figure 2:**
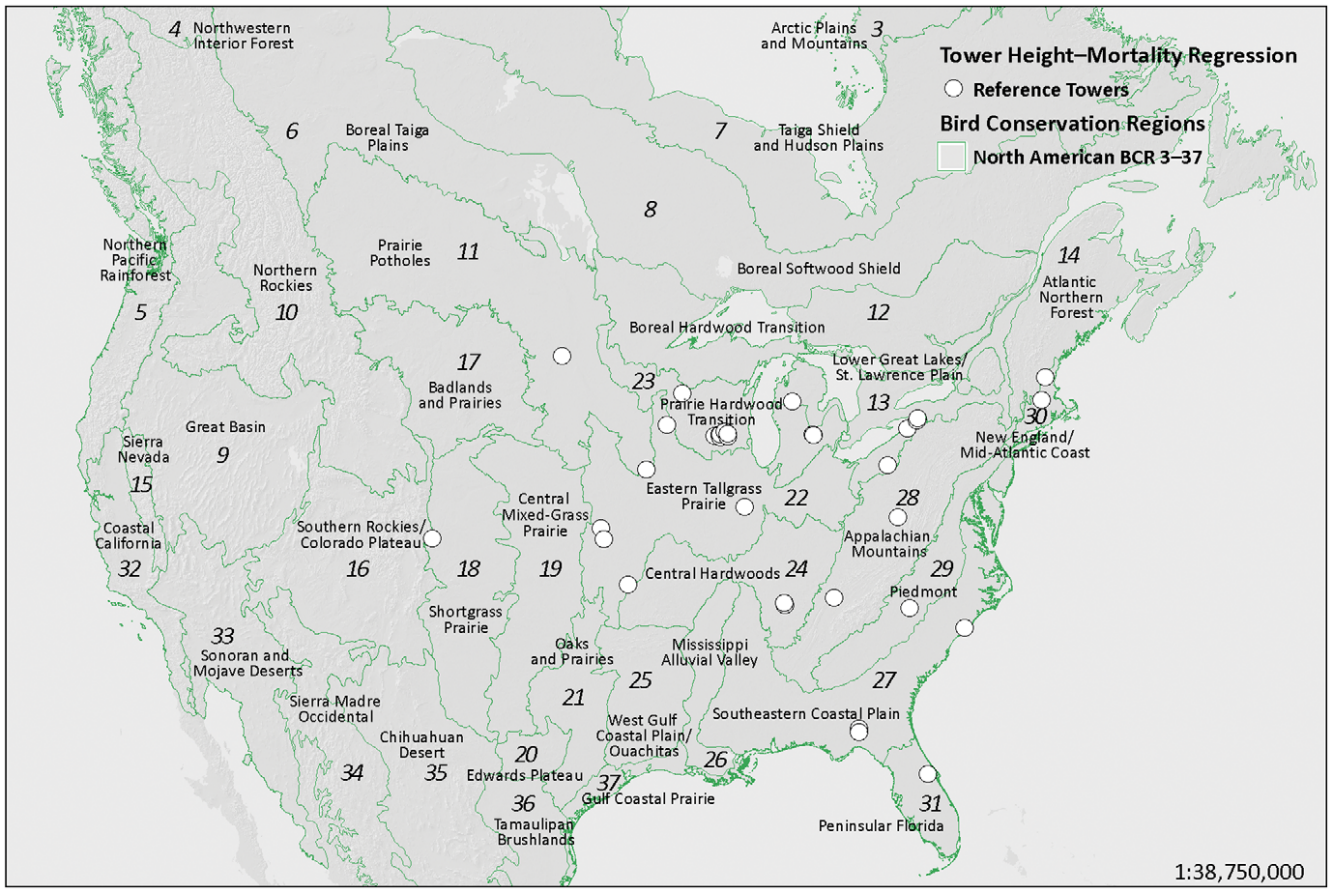
Bird Conservation Regions and locations of towers used for tower height–mortality regression.

**Figure 3:**
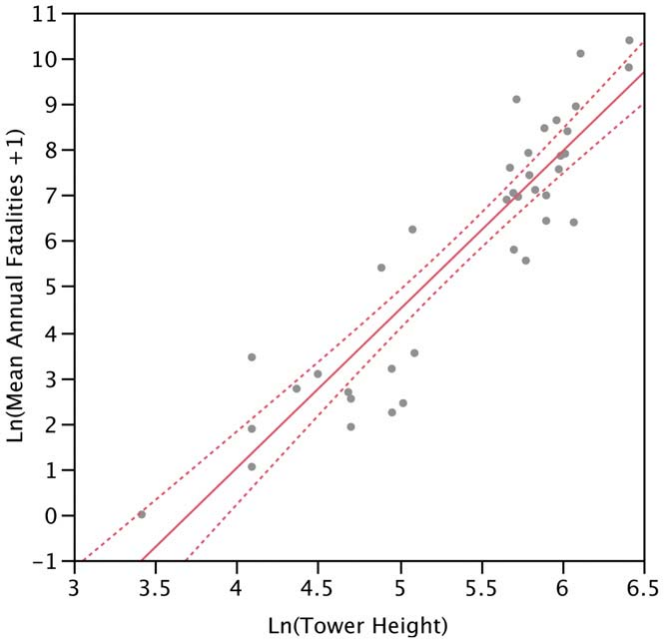
Regression and 95% confidence intervals of annual avian fatalities by tower height. Annual avian fatalities were adjusted for sampling effort, search efficiency, and scavenging and regressed by log-transformed tower height (Ln(Mean Annual Fatalities +1) = 3.4684 · Ln(Tower Height) – 12.86, *R* ^2^ = 0.84, p<0.0001).

Towers used in the height–mortality regression were located throughout the eastern United States (Figure 2). We were able to confirm from original sources and personal communications that 68.4% of the towers were chosen for study with no prior knowledge of avian mortality; status is unknown for 13.2% of towers; and only 18.4% of towers were chosen with any knowledge of prior avian mortality. Log-transformed annual avian mortality, when adjusted for sampling effort, search efficiency, and scavenging, was significantly explained by log-transformed tower height in a linear regression (*R* ^2^ = 0.84, F_1,36_ = 191.62, p<0.0001) (Table 5; Figure 3).

**Table 5:** Regression results for mean annual fatalities by tower height, when unadjusted, corrected for sampling only, corrected for search efficiency and scavenging only, and corrected for both sampling and search efficiency/scavenging, with estimated annual fatalities after back transformation, adjustment for bias, and application to all towers in the United States and Canada.

	Slope	Intercept	R^2^_adj_	RMSE	F	P	Estimated annual fatalities (million)
No corrections	2.8257	–10.8626	0.78	1.110	133.5046	<0.0001	1.38
Sampling correction	3.0962	–11.9490	0.80	1.151	148.8302	<0.0001	2.06
Searcher/scavenging correction	3.2024	–11.8012	0.82	1.110	171.2329	<0.0001	4.31
Both corrections	3.4684	–12.8600	0.84	1.137	191.6163	<0.0001	6.80

### Tower Height–mortality Regression Sensitivity to Study Inclusion

**Figure 4:**
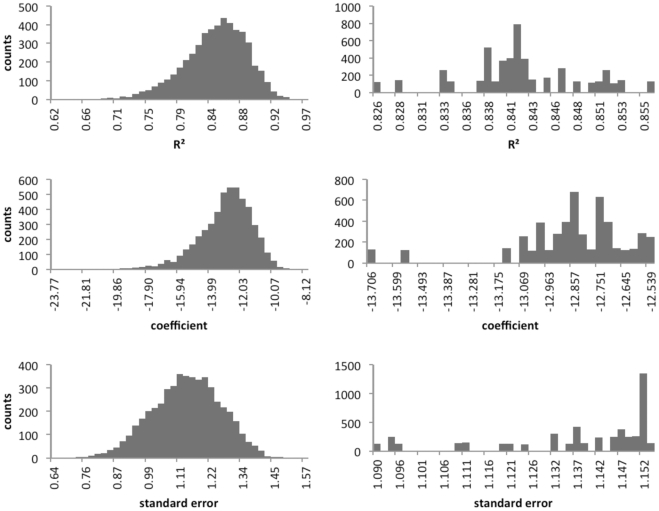
Influence of study choice on tower height–mortality regression. Distribution of counts for *R* ^2^ (adjusted), standard error, and coefficient for 5,000 iterations (subset = 18 studies, left; subset = 37 studies, right) for a linear regression between the natural logarithms of tower height (m) and mean annual fatalities.

The median *R* ^2^ values of the resampled distributions are similar to those obtained from using all of the available studies (Figure 4, Table 6) and are not sensitive to the addition or elimination of a few or a set of studies. The results of the resampling procedure for subsets of 18 studies (a little under half of the studies) and for 37 studies (1 fewer than the total) show the range of influence that study inclusion could have on the regression line (Table 6).

**Table 6:** Confidence intervals and median values for model parameters using randomized subsets of 18 or 37 studies (5,000 iterations).

Subset	Parameter	5%	95%	Median
***18 studies***	*R* ^2^	0.765	0.906	0.847
	slope	3.087	4.061	3.474
	intercept	–16.205	–10.775	–12.882
	standard error	0.919	1.331	1.345
***37 studies***	*R* ^2^	0.828	0.853	0.841
	slope	3.414	3.591	3.465
	intercept	–13.556	–12.556	–12.845
	standard error	1.093	1.153	1.146

### Evaluation of Model Adjustment Factors

Models using either sampling correction alone or the combination of sampling correction with the combined search efficiency and scavenging correction were found to be superior to the model using tower height alone at explaining annual kills (*R* ^2^ = 0.84 vs. *R* ^2^ = 0.79; Table 5). Correcting for search efficiency and scavenging losses appeared to provide the best improvement to the overall model (Table 5).

### Tower Characteristics

**Figure 5:**
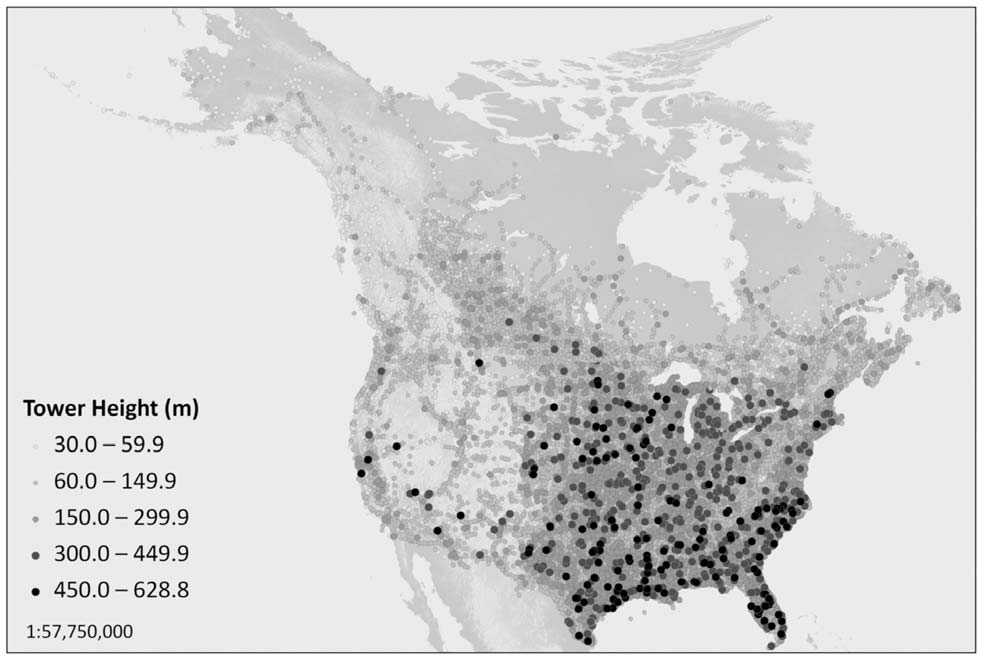
Map of communication towers in the United States and Canada by height class. Data acquired from Federal Communications Commission, Towermaps.com, and NAV CANADA.

Our database of ≥60 m towers included 70,414 towers in the continental United States and Canada after all quality assurance and quality control was done (Figure 5). Most towers in the United States dataset (31,486; 50.3%) were freestanding with steady-burning lights at night, while the fewest towers (4,898; 7.8%) were guyed with strobe lights at night. Some towers had strobe lights during the day but red flashing and red solid lights at night so these were included as having solid lights.

### Total Mortality and Estimates by Bird Conservation Region

Combination of the tower height–mortality regression with estimates of reduced mortality at towers without guy wires or steady-burning lights produced a matrix of mortality by height class and tower characteristics. These estimates, already adjusted for sampling effort, search efficiency, and scavenging, ranged from zero for short unguyed towers to over 20,000 birds per year for the tallest guyed towers with steady-burning lights.

The back-transformed tower height–mortality regression, adjusted for bias (smearing estimator) and applied to towers in the continental United States and Canada, produced an annual mortality estimate of 6.8 million birds per year (Table 5). Extrapolation from the unadjusted data yielded an estimate of 1.4 million birds per year, meaning that our cumulative assumption is that searchers find only around 20% of the birds that are killed, because of search efficiency, scavenging, and incomplete sampling (Table 5).

These results are sensitive to the assumptions that were made about these factors. As an illustration, we calculated total mortality while assuming a constant search efficiency equal to the average of the measured search efficiency from those towers where this was measured (36.4%), which resulted in a total mortality estimate of 9.4 million birds per year. Applying the average scavenging rate (15.8%) to all towers resulted in a mortality estimate of 4.7 million birds per year. Using both averages (for scavenging and search efficiency) yielded an estimate of 6.4 million birds per year. For the sampling effort adjustments, recalculated mortality estimates for the three scenarios applied to studies with unknown sampling schemes were: 5.4 million birds per year for sampling only on big kill days, 5.7 million birds per year for sampling on bad weather days and weekly year round, and 6.2 million birds per year for sampling on bad weather days and weekly during migration only. Finally, if we recalculate mortality after omitting all towers selected with prior knowledge of any mortality on site (18.4% of our sample of towers), the estimate of total mortality declines to 5.5 million birds per year.

Over two-thirds of the estimated mortality is attributed to towers ≥300 m, of which only 1,040 were found in our database (1.6% of towers ≥60 m; Table 7). Fully 71% of mortality is attributed to the tallest 1.9% of towers. Shorter towers (60–150 m) contribute approximately 17% of all mortality because of their sheer numbers (Table 7).

**Table 7:** Number of communication towers ≥60 m by type and associated avian mortality estimates for Canada and the continental United States.

Country	Height class (m)	Guyed towers with steady-burning lights	Guyed towers with strobe lights	Unguyed towers with steady-burning lights	Unguyed towers with strobe lights	Annual fatalities	Percent of fatalities
***United States***	60–90	5,901	863	17,693	2,575	115,524	1.76%
	90–120	10,023	1,696	10,004	1,683	531,411	8.07%
	120–150	2,938	505	2,922	488	377,542	5.74%
	150–180	1,992	311	661	101	468,600	7.12%
	180–210	343	46	107	12	142,679	2.17%
	210–240	174	54	51	11	126,507	1.92%
	240–270	109	57	29	16	131,379	2.00%
	270–300	76	61	18	14	146,530	2.23%
	300–330	271	128	0	0	642,858	9.77%
	330–360	115	28	0	0	345,255	5.25%
	360–390	78	22	0	0	317,130	4.82%
	390–420	47	16	0	0	254,809	3.87%
	420–450	35	10	0	0	238,450	3.62%
	450–480	66	23	0	0	579,458	8.80%
	480–510	25	10	0	0	277,580	4.22%
	510–540	24	8	0	0	319,300	4.85%
	540–570	8	9	0	0	165,120	2.51%
	570–600	18	15	0	0	410,068	6.23%
	600–630	38	27	0	0	991,745	15.07%
	*Subtotal*	*22,282*	*3,888*	*31,486*	*4,898*	*6,581,945*	*100.00%*
***Canada*** 1	60–90	627	323	1,880	968	13,980	6.34%
	90–120	1,295	284	1,295	284	69,981	31.72%
	120–150	251	55	251	55	32,797	14.86%
	150–180	92	23	31	8	22,363	10.14%
	180–210	44	11	15	4	19,085	8.65%
	210–240	19	5	6	2	13,757	6.24%
	240–270	6	2	2	1	6,640	3.01%
	270–300	3	1	1	0	4,884	2.21%
	300–330	9	4	0	0	21,267	9.64%
	330–360	3	1	0	0	8,973	4.07%
	360–390	1	0	0	0	2,996	1.36%
	390–420	1	0	0	0	3,912	1.77%
	*Subtotal*	*2,349*	*709*	*3,480*	*1,321*	*220,650*	*100.00%*
***Total***		***24,631***	***4,597***	***34,966***	***6,219***	***6,802,595***	

1 Tower attributes (guy wires, lighting type) for Canada are extrapolated from proportions in the United States because these attributes are not found in the NAV CANADA database.

**Figure 6:**
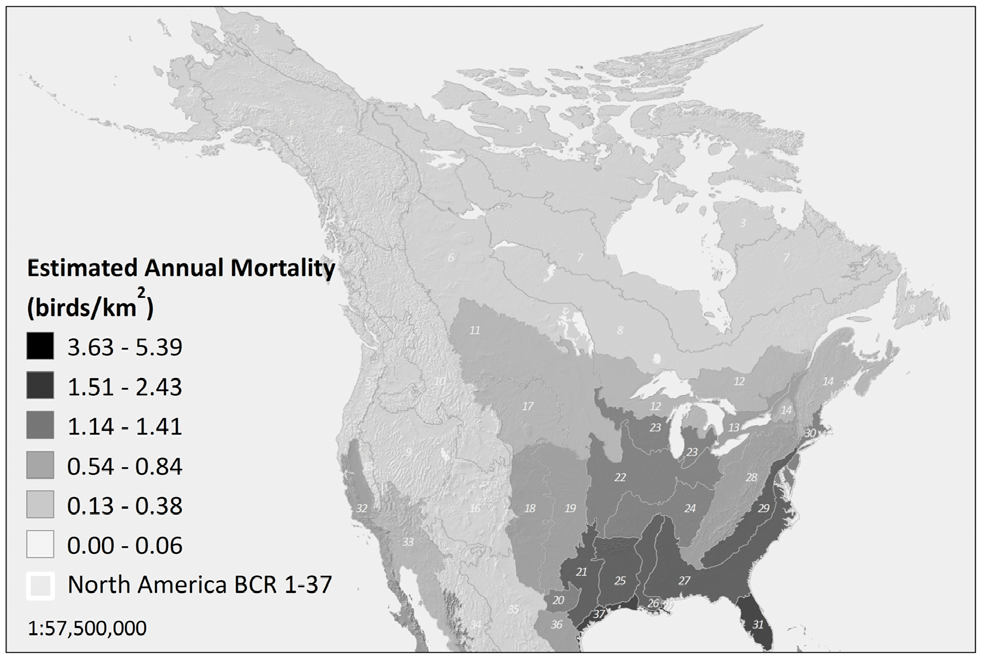
Estimated annual avian mortality from communication towers by Bird Conservation Region. High mortality estimates in Peninsular Florida and Southeastern Coastal Plain reflect the more numerous and taller communication towers in these regions.

Our estimates of mortality vary by region, influenced both by the size of the region and the number and height distribution of towers (Figure 6; Table 8). The number of towers in each BCR does not directly correlate with estimated annual mortality because of differing numbers and heights of towers. As a result, Peninsular Florida is associated with more mortality than all of Canada; even though fewer towers are reported in Peninsular Florida, they are on average much taller. The concentration of migrants resulting from Florida’s geographic position would increase mortality even more, but this factor is not considered in our method because mortality rates for any given tower height are assumed to be constant across the continent. The Southeastern Coastal Plain BCR accounts for greater mortality than other BCRs, followed by Eastern Tallgrass Prairie, Oaks and Prairies, and Piedmont (Table 8). Canadian mortality accounts for only a fraction of the total (approximately 3.2%), because Canada has far fewer, and generally shorter, towers.

**Table 8:** Total estimated annual avian mortality at towers ≥60 m in the United States and Canada by Bird Conservation Region (BCR).

BCR	USA (lower 48 states)	Canada	Alaska	Total
1–Aleutian Bering Sea			0	0
2–Western Alaska			155	155
3–Arctic Plains and Mountains		542	83	625
4–Northwestern Interior Forest		288	2,228	2,516
5–Northern Pacific Rainforest	21,170	2,411	333	23,914
6–Boreal Taiga Plains		24,591		24,591
7–Taiga Shield and Hudson Plains		2,754		2,754
8–Boreal Softwood Shield		20,650		20,650
9–Great Basin	20,744	339		21,083
10–Northern Rockies	8,653	1,925		10,578
11–Prairie Potholes	265,244	63,032		328,276
12–Boreal Hardwood Transition	139,535	34,564		174,099
13–Lower Great Lakes/St. Lawrence Plain	83,185	51,175		134,360
14–Atlantic Northern Forest	36,469	18,378		54,847
15–Sierra Nevada	343			343
16–Southern Rockies/Colorado Plateau	29,175			29,175
17–Badlands and Prairies	54,040			54,040
18–Shortgrass Prairie	243,791			243,791
19–Central Mixed-Grass Prairie	333,211			333,211
20–Edwards Plateau	81,827			81,827
21–Oaks and Prairies	469,889			469,889
22–Eastern Tallgrass Prairie	754,928			754,928
23–Prairie Hardwood Transition	278,788			278,788
24–Central Hardwoods	346,796			346,796
25–West Gulf Coastal Plain/Ouachitas	321,983			321,983
26–Mississippi Alluvial Valley	185,746			185,746
27–Southeastern Coastal Plain	1,107,118			1,107,118
28–Appalachian Mountains	263,368			263,368
29–Piedmont	448,533			448,533
30–New England/Mid-Atlantic Coast	96,197			96,197
31–Peninsular Florida	341,774			341,774
32–Coastal California	99,873			99,873
33–Sonoran and Mojave Deserts	50,179			50,179
34–Sierra Madre Occidental	875			875
35–Chihuahuan Desert	16,559			16,559
36–Tamaulipan Brushlands	105,545			105,545
37–Gulf Coastal Prairie	373,609			373,609
***Total***	***6,579,147***	***220,649***	***2,799***	***6,802,595***

**Figure 7:**
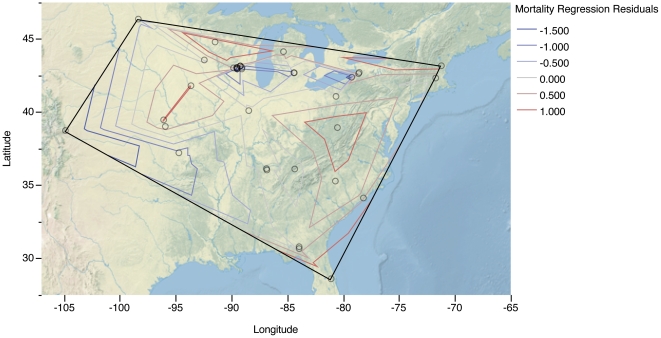
Distribution of residuals of tower height–mortality regression for 38 towers in the United States as adjusted for sampling effort, search efficiency, and scavenging. Contour lines indicate regions above and below the regression line. Although exhibiting a geographically variable pattern, the residuals are not significantly spatially autocorrelated.

Although we extended mortality estimates to all towers in Canada and the continental United States, few studies are available from the West (Figure 2). This may be a function of a higher number of nocturnal migrants in the East, different patterns of migration, different weather patterns, or it may simply reflect the fewer and shorter towers in the West as a whole. We investigated the effect of location on annual mortality by regressing the residuals of our height regression against longitude and also by testing the residuals for spatial autocorrelation. The resulting plot showed slightly higher mortality in the East, but the relationship was not significant and was largely driven by a single data point in Colorado. Residuals were not spatially autocorrelated using inverse Euclidean distance weighting (Figure 7; Moran’s I = 0.09, z = 0.23, *p* = 0.816). More comprehensive surveys of towers in the West are needed to see if the lower mortality at the site in Colorado represents an anomaly or a different pattern of mortality in the West. Pending such further analysis, extrapolation of mortality at towers in the western portions of the United States and Canada should be regarded as provisional.

## Discussion

Our total mortality estimate of 6.8 million birds per year is ∼50% greater than the current USFWS estimate of 4–5 million birds per year [14], [15], [23], [24]. Our results do not support the suggestion that mortality might be an order of magnitude higher [14], [15], which had been made before this type of synthetic analysis had been attempted. Our approach to estimating total avian mortality at towers uses far more data than previous efforts. For example, Banks’s [13] estimate was based on mortality rates from only three tower studies and assumed that all towers caused the same rate of mortality, regardless of tower height. Our method incorporates evidence from 38 towers to establish the relationship between tower height and avian mortality. We accounted for the height distribution and physical characteristics of ∼84,000 towers across the United States and Canada (including towers <60 m, which we mapped but did not include in our mortality estimates). Notwithstanding the sources of uncertainty in our estimate, the method improves previous efforts, is transparent, and can be revised in conjunction with additional field studies.

Although mortality at some towers has apparently declined over time [31], the influence of any such trend (if a true decline in mortality and not the result of increased scavenging) is offset by the large portion (>50%) of towers in the regression having survey end dates after 1990. If only these studies ending after 1990 are used in the regression, the total mortality estimate decreases to 4.8 million birds per year. The residuals of the tower height–mortality regression, however, are not significantly explained by the ending year of the survey (results not shown) so we did not exclude the older studies from our final regression. Even if the decline in number of birds killed at towers is a real phenomenon, the effect of these kills on sensitive species could still be substantial if populations have declined by a greater proportion.

Estimated tower mortality increases exponentially with tower height [26], which makes our results sensitive to the use of the height classes. For example, if we used the top of each height class rather than the middle to calculate total mortality, the estimate would increase by 25%. The use of the height classifications was necessary for ease of calculation and because attributes of the Canadian towers that were not known had to be assigned probabilistically. We used log transformations of both variables to normalize the distributions and because the total volume of airspace occupied by guy wires increases far more rapidly than does height. The increasing length of guy wires provides a mechanistic explanation for the exponentially increasing probability of avian collisions as tower height increases. Extremely tall towers also extend into the “normal” flight altitudes of many migrants so that mortality events can occur under clear skies and favorable migration conditions; this provides another plausible mechanism for the exponential increase in mortality rates observed by height. We also considered using separate regressions for towers less than and greater than 200 m, given the break in the data, but found that doing so had little effect on the overall estimate and we could not formulate a functional explanation why the tower height–mortality relationship should change in this manner.

Further research is needed on the mortality rates at the tallest towers (i.e., >500 m). These data are needed to confirm that the tower height–mortality relationship is exponential [26]. The nature of this relationship is important because it leads directly to a policy recommendation of focusing on the tallest towers first for mitigation. If more extensive tower datasets show a different relationship (e.g., logistic) then mitigation actions would be much different, requiring treatment of many more towers to address the same proportion of mortality.

Producing this estimate of avian mortality at towers required many assumptions, the implications of which we have explored to the degree possible with the data available. By undertaking this exercise, we have reaffirmed what elements should be included in tower studies going forward – explicit measurement of search efficiency, scavenging rates, and the effect of sampling schemes for any study, as well as investigation of geographic variation in mortality and inclusion of towers representative of the extremes of the height distribution. Such research will help refine our regionalized mortality estimates.

In 1989, the Exxon Valdez oil spill killed approximately 250,000 birds in what has become the benchmark for a major environmental disaster [65]. Our estimates show that communication towers are responsible for bird deaths equivalent to more than 27 Exxon Valdez disasters each year. Our estimate of the number of birds killed annually by communication towers is 2–4 times greater than the estimate for annual fatalities from lead poisoning before lead shot was phased out for hunting waterfowl [66]. Previous efforts (e.g., [25]) and our compiled database illustrate that most of the birds killed at communication towers are Neotropical migrants, which have suffered population declines and many of which are formally recognized as “Birds of Conservation Concern” [67], [68]. Data on per species mortality would provide even more clarity about the biological significance of avian mortality at communication towers. In a companion manuscript, we estimate species-specific losses based on total losses estimated here and species-specific casualty reports for Bird Conservation Regions following methods we developed previously [35]. But even without such estimates, the aggregate mortality numbers developed here should lead policymakers to pursue mitigation measures to reduce this source of chronic mortality.

Mitigation of avian mortality at communication towers could most practicably be achieved by implementing several measures: 1) concomitant with permission from aviation authorities, remove steady-burning red lights from towers, leaving only flashing (not slow pulsing) red, red strobe, or white strobe lights [24], [26], [28], [31]; 2) avoid floodlights and other light sources at the bases of towers, especially those left on all night [64]; 3) avoid guy wires where practicable [26], [28]; 4) minimize the number of new towers by encouraging collocation of equipment owned by competing companies; and 5) limit height of new towers when possible. Concentrating on removing steady-burning lights from the roughly 4,500 towers ≥150 m tall in the United States and Canada with such lights should be a top priority because, according to our model, it would reduce overall mortality by approximately 45% through remedial action at only 6% of lighted towers.
